# Patient-related effects of primary nursing: Protocol of a pilot randomized controlled trial

**DOI:** 10.1007/s00063-023-00998-w

**Published:** 2023-03-27

**Authors:** Lars Krüger, Thomas Mannebach, Armin Zittermann, Franziska Wefer, Vera von Dossow, Sebastian Rojas Hernandez, Jan Gummert, Gero Langer

**Affiliations:** 1 Project and Knowledge Management/Care Development intensive care, Care Directorate, Heart and Diabetes Center NRW, University Hospital of the Ruhr University Bochum, Georgstraße 11, 32345 Bad Oeynhausen, Germany; 2 Surgical Intensive Care Unit E 0.1, Heart and Diabetes Center NRW, University Hospital of the Ruhr University Bochum, Georgstraße 11, 32345 Bad Oeynhausen, Germany; 3 Clinic for Thoracic and Cardiovascular Surgery, Heart and Diabetes Center NRW, University Hospital of the Ruhr University Bochum, Georgstraße 11, 32345 Bad Oeynhausen, Germany; 4 Care Development, Care Directorate, Heart and Diabetes Center NRW, University Hospital of the Ruhr University Bochum, Georgstraße 11, 32345 Bad Oeynhausen, Germany; 5 Institute for Nursing Science, Medical Faculty and University Hospital Cologne, University of Cologne, Gleueler Straße 176–178, 50935 Cologne, Germany; 6 Institute of Anesthesiology and Pain Therapy, Heart and Diabetes Center NRW, University Hospital of the Ruhr University Bochum, Georgstraße 11, 32345 Bad Oeynhausen, Germany; 7 Institute of Health and Nursing Sciences, German Center for Evidence-based Nursing, Martin Luther University Halle-Wittenberg, Magdeburger Straße 8, 06112 Halle (Saale), Germany

**Keywords:** Anxiety, Delirium, Intensive care units, Nursing process, Relatives, Angehörige, Angstzustände, Delir, Intensivstation, Pflegeprozess

## Abstract

**Background:**

Since January 2022, a primary nursing system called process-responsible nursing (PP) has substituted the standard room care system in an intensive care unit (ICU) at our institution. The process of the development and implementation of PP is already being evaluated in a separate study as an actual analysis prior to implementation, as well as after 6 and 12 months.

**Aim:**

This pilot randomized controlled trial (RCT) aims to test the feasibility of an RCT. For this purpose, the duration of delirium, among other things, will be compared in the project ICU with the results of standard care in another ICU at the university hospital. As secondary aims, the incidence of delirium, anxiety, the satisfaction of relatives, and the effects of PP on nurses will be assessed.

**Methods:**

It is planned to recruit about 400–500 patients over a period of one year. They will be allocated to PP or standard care. Delirium will be assessed using the Confusion Assessment Method for Intensive Care Units by specifically trained nurses three times a day. Anxiety in patients, the satisfaction of relatives, and the effects of PP on nurses will be evaluated using the numeric rating scale, a standardized questionnaire, and a focus group interview, respectively.

**Expected results:**

The primary hypothesis is that compared to usual care PP reduces the duration of delirium by at least 8 h. Additional hypotheses are that PP reduces anxiety in patients and increases the satisfaction of relatives.

**Supplementary Information:**

The online version of this article (10.1007/s00063-023-00998-w) includes the SPIRIT 2013 Checklist.

## Abstract (de)

**Hintergrund:**

Seit Januar 2022 wird in unserer Einrichtung auf einer Intensivstation (ITS) ein Bezugspflegesystem, die sog. prozessverantwortliche Pflege (PP) anstelle der Zimmerpflege umgesetzt. Die Entwicklung und Implementierung von PP wird in einer separaten Studie als Ist-Analyse vor sowie 6 bzw. 12 Monate nach Umsetzung evaluiert.

**Ziel:**

Ziel dieser randomisierten kontrollierten Pilotstudie (RCT) ist es, die Durchführbarkeit einer RCT zu testen. Dazu wird die Dauer des Delirs auf der Projekt-ITS mit den Ergebnissen der Standardpflege auf einer anderen ITS des Universitätsklinikums verglichen. Als sekundäre Ziele werden das Auftreten von Delirien, Angstzustände, die Zufriedenheit der Angehörigen und die Auswirkungen von PP auf die Pflegenden untersucht.

**Methoden:**

In dieser Pilot-RCT werden ca. 400–500 potenzielle Patientinnen und Patienten über ein Jahr der PP oder der Standardpflege zugeteilt. Das Auftreten eines Delirs wird dreimal täglich von speziell geschulten Pflegefachpersonen mithilfe der Confusion Assessment Method for Intensive Care Units beurteilt. Ängste der Patientinnen und Patienten, Zufriedenheit der Angehörigen und die Auswirkungen von PP auf die Pflegenden werden anhand einer numerischen Bewertungsskala, eines standardisierten Fragebogens bzw. eines Fokusgruppeninterviews untersucht.

**Erwartete Ergebnisse:**

Die primäre Hypothese ist, dass PP im Vergleich zur Standardpflege die Dauer des Delirs um mindestens 8 h verkürzt. Weitere Hypothesen sind, dass PP die Angst der Patientinnen und Patienten reduziert und die Zufriedenheit der Angehörigen erhöht.

## Background

More than 50 years ago, Manthey et al. [33] developed a nursing system called primary nursing (PN). PN consists of four basic elements: personal responsibility for decision-making and the acceptance of that by one person, daily work assignment according to the case method, direct communication between all parties involved, and finally taking responsibility for the quality of care of the assigned patients over 24 h and 7 days per week [33].

In recent years, data from studies on PN have been published in various articles. Results include an improvement in the quality of care [5] by improved direct communication by nurses [35, 45] and an increasing orientation of nurses to the needs of patients [4, 16, 17, 29]. In addition, reductions in the incidence of catheter infections (urinary and central venous catheters) and pressure ulcers were reported [9]. Furthermore, the introduction of PN was associated with positive effects on increasing professional autonomy [34], a possibly decreasing incapacity for work [5], and on nursing responsibilities [18].

Fröhlich et al. [17] evaluated the implementation of PN in three ICUs in a university hospital in Zurich, Switzerland. The authors showed that “Bezugspflege” was implemented as a synonym for PN. Based on a cross-sectional study conducted in the same facility, Naef et al. [37] found in 2017 that the surveyed patients reported a high quality of individualized, responsive and competent care, but only a lower level of coordinated care. Most nurses reported that PN is beneficial for person-centered care but was not fully practiced consistently in their unit [37]. Rebitzer [40] evaluated the introduction of PN in a cardiothoracic vascular surgical ICU in Austria by using primarily qualitative methods as part of a doctoral thesis. In summary, positive effects of interdisciplinary cooperation and optimization of nursing documentation could be determined.

Nevertheless, in a review by Butler et al. [6], no substantial improvements in patient outcomes could be identified after introducing PN because there was a lack of research. However, in that review, the results of Dal Molin et al. [9] were not included. Likewise, Butler et al. [6] only found low evidence regarding the turnover of nurses and cost reduction in hospitals by PN.

Concerning delirium, Eckstein and Burkhardt [13] were among the first to identify a possible positive impact of PN in hospital settings for nonpharmacological interventions. The incidence of delirium in cardiac surgery ranges from < 10% [20] to > 25% [8, 27]. Different pharmacological, but also nonpharmacological measures can positively counteract the development of delirium [23, 24]. These include, for example, adherence to the day–night rhythm [11, 22] and avoiding noise [10, 11]. Data regarding the effects of PN on planning, performance, and evaluation of nonpharmacological delirium prevention/intervention are scarce.

To close this gap, a comparative study between a project surgical ICU, in which PP intervention is implemented, and another surgical ICU, in which standard care is executed, is to be carried out as a pilot randomized controlled trial (pilot-RCT) to test the feasibility of an RCT [14, 44]. In addition, we will study the effect of PN on the duration and incidence of delirium, as well as the satisfaction of relatives with patient care.

## Methods

### Study design and setting

This study report follows the recommendations for reporting study protocols of clinical intervention studies (Standard Protocol Items: Recommendations for Interventional Trials, SPIRIT [7]). The SPIRIT 2013 Checklist was used and is published as a supplementary file in the online version of this article. It is a pilot-RCT that will be carried out at the Heart and Diabetes Center (HDZ) North Rhine–Westphalia (NRW), university hospital of the Ruhr University Bochum, Germany, on a surgical project ICU (ICU1) and another surgical ICU (ICU2). The two ICUs are in different parts of the building and belong to the Clinic for Thoracic and Cardiovascular Surgery. Patients will be randomized to the intervention or control group. Patients in the intervention and control groups will be treated in the aforementioned two different ICUs according to the PP principle or the principles of standard care, respectively.

**Fig. 1:**
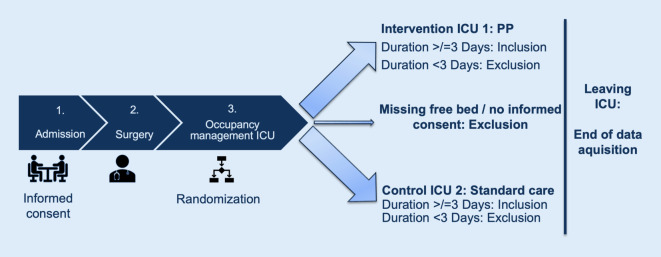
Course of the study

For study participation, written and informed consent is required. The ethics committee of the medical faculty of the Ruhr University Bochum, based in East Westphalia, has already approved the study (file number 2022-952). In addition, the study had been registered at ClinicalTrials.gov as NCT05569317. The course of the study is shown in Fig. 1.

### Participants

Patients are eligible for study participation if they (i) underwent elective cardiac surgery, (ii) are 65 years of age or older, (iii) are familiar with the German language, (iv) give their informed consent, and (v) their health insurance company has concluded a special quality contract with HDZ NRW. In addition, the expected ICU stay should be at least 3 days. Exclusion criteria are the following: a patient age < 65 years, a lack of vacant beds in the destination ICU, missing informed consent, and the impossibility of participation in the neurological screening with the Confusion Assessment Method for Intensive Care Units (CAM-ICU) due to neurological diseases. This trial plans to include 400–500 patients and their relatives.

A total of six nurses from the ICU 1 will be included in the trial for a focus group interview. To be eligible for study participation, nurses and patient relatives must be at least 18 years of age.

### Recruitment, randomization, and treatment allocation

Patients and relatives will be recruited ad hoc during elective admission and treatment at HDZ NRW between January and December 2023. Study invitations will be performed by trained (nursing) specialists. Patients will be assigned to the intervention (ICU 1) or control ICU (ICU 2) post surgery by block randomization. Randomization will be based on blocks with 6 patients in each block, generated by using the random function of Microsoft Excel 2019 (Microsoft, Redmond, WA, USA). The intervention group will be allocated to a so-called process-responsible care system (PP). Briefly, in the PP system, process responsible nurses (also called PP) take on the responsibility of the nursing process, the daily care according to the individual patient case, direct communication with all professional groups involved in the care process, and responsibility for the quality of care of the assigned patients over a fixed period [33] of at least 14 and maximum 50 days. After that, the PP will change.

In this way, PP also take on the nursing anamnesis and the planning, implementation and evaluation of nursing care. The nurse-to-patient ratio will be at least 1:2. PP plan, coordinate and evaluate the activities of the various professional groups in patient care, such as physicians, physiotherapists, psychologists or speech therapists. Together with this interdisciplinary treatment team, the inpatient course of the patient will be controlled in a targeted manner [33]. The procedure is synonymous with the internationally used term PN [33]. These tasks also include the reserved activities of nurses in Germany [39]. Nurses who have completed a bachelor’s degree in nursing or a German state-approved training course in intensive and anesthesia nursing care can become PP nurses. In addition, completed training within the surgical intensive care units in the HDZ NRW, and at least 3 years of professional experience in these ICUs are required. Temporary specific further training in PP is also possible for nurses who have been trained for 3 years (registered nurses). An actual analysis was carried out as a starting point for the PP project development at HDZ NRW, which follows the recommendations of the Medical Research Council (MRC) [42]. In addition, an evaluation after the introduction of PP is planned after 6 and 12 months of implementation. The data were collected within a separate study (“Analysis of process-responsible care”), which was registered in the German Register for Clinical Studies (DRKS-ID: DRKS00024612) and ended in December 2022.

The control group with patients who are cared for according to the principles of standard care (room care or area care) will be located in another ICU (ICU 2). There will be no PP responsible for the target group, and nursing staff allocation to patients will be redefined at the start of each shift. The nurse-to-patient ratio will be at least 1:2.

During a visit to the hospital, the relatives of the patients will be personally informed by the study management or trained (nursing) professionals and invited to participate in the study. Likewise, the nursing staff will be personally invited by the study management or trained (nursing) specialists to take part in the study. Their written consent will be obtained before a focus group interview starts.

### Blinding

This is an open-labeled study and there will be no blinding. Nurses in the intervention and control units cannot be blinded to the care they provide. Blinding patients about the care they receive is also not possible. Especially trained nurses who carry out the data acquisition cannot be blinded either, since they perceive the implemented care concept when entering the respective units through their nursing expertise.

Study bias through the monocentric implementation can be reduced to a limited extent because the involved ICUs are spatially separated within the clinic in two different parts of the building and are also cared for by separate nursing teams.

### Endpoints

The primary aim of this pilot-RCT is to test the feasibility of an RCT regarding patient-related outcomes [3, 46] after the implementation of PP in ICU 1 compared with those of standard care in ICU 2 at HDZ NRW. For this purpose, the percentage of recruited patients (recruitment rate) and the number of patients who received PP or standard care (delivery rate) will be measured. The primary clinical endpoint is the duration of delirium. Secondary endpoints are the incidence of delirium, pain, anxiety, the incidence of pressure ulcers, the need for care, the satisfaction of the relatives and the impact of PP on nursing staff. The main research question is: What effects does the implementation of PP in the ICU have on the duration of delirium in patients ≥ 65 years after elective thoracic and cardiovascular surgery? The following primary hypothesis was formulated: PP reduces the duration of delirium in patients ≥ 65 years after elective thoracic and cardiovascular surgery by at least 8 h compared to standard care. Additional hypotheses were formulated as follows: PP reduces anxiety in patients ≥ 65 years after elective thoracic and cardiovascular surgery and PP increases the satisfaction of relatives compared to standard care.

### Statistical methods

The duration of delirium will be given as mean with standard deviation (SD), mean difference and confidence interval in case of normally distributed data. Otherwise, data will be presented as a median with interquartile range (IQR) and confidence interval.

For group comparison, the unpaired t‑test will be used for normally distributed data and the Mann–Whitney U‑test for nonnormally distributed data. A test for normal distribution will be carried out using the Kolmogorov–Smirnov test.

Secondary endpoints will be evaluated using methods of descriptive statistics and, if possible, compared with mean values, effect sizes and the associated measures of dispersion and interdependence tests. The percentage of recruited patients (recruitment rate) and the number of patients who received PP or standard care (delivery rate) will be stated descriptively.

### Instruments/data collection

Data will be recorded in a pseudonymized manner. References to individuals will thus be replaced by a numerical code in all files used in the study evaluation. However, if necessary, this number code can be used to trace back the identity of the person concerned.

#### Incidence and duration of delirium and pain

The incidence and duration of delirium and pain will be recorded via daily monitoring, once per shift in a three-shift system, using validated assessment instruments. This includes the Confusion Assessment Method for Intensive Care Units (CAM-ICU) [1, 19] as a validated assessment for, e.g., nurses to identify delirium in patients in combination with the Richmond Agitation Sedation Scale (RASS) [15, 41], the numeric rating scale (NRS) [28] or, if the use of NRS is not possible, the Critical Care Pain Observation Tool (CPOT) [30]. The first survey will take place upon admission to HDZ NRW.

The duration of delirium will be reported in blocks of 8 h. The end of delirium is determined when 3 consecutive data recordings with the CAM-ICU are negative, and, thus, no more delirium has been detected for a total of 24 h.

#### Anxiety

Anxiety is measured by using the validated instrument of the numeric rating scale (NRS) [2, 28] if the patient is able to use the NRS. The first survey takes place upon admission to HDZ NRW. Further surveys take place once per shift after admission to the ICU.

#### Incidence of pressure ulcer

The incidence of pressure ulcers in the included patients will be read out from the patient data management system (PDMS) documentation. In addition, the Braden scale will be used to assess the risk of pressure ulcers [21]. The first survey will take place upon admission to HDZ NRW. The further surveys will take place once a day. The Braden scale will be filled out by trained nursing staff during night shifts.

#### Need for care

The Barthel Index will be used to measure the need for care [25]. The result of the assessment will be read out via the documentation of the PDMS. The first survey will take place upon admission to HDZ NRW. The further surveys will take place once a week.

#### Satisfaction of the relatives

The satisfaction of relatives will be surveyed with the questionnaire of Huber et al. [26], which was developed for the measurement of patient satisfaction in geriatric units of hospitals. The survey will be carried out once during the stay in the ICU.

#### Effects of PP on nurses

The effects of PP on nurses will be determined using a focus group interview with six nurses. For this purpose, specific openly formulated questions have been developed. The results will be placed in context with the data from the development process of PP (DRKS-ID: DRKS00024612) and evaluated using the method of content-structuring qualitative content analysis according to Kuckartz [32]. Data collection will take place in the third to fourth quarter of 2023.

## Discussion

This pilot study can make an important contribution to investigating the effects of PP on patients, their relatives, and nurses in ICUs in Germany. The burden and risks for the participants can be classified as very low since data recording is a regular part of the daily monitoring of professional nurses in the ICU.

To the best of our knowledge, similar studies are almost completely lacking. Thus, a reasonable power calculation is not possible. Under the assumption that PP is comparable with the effects of nonpharmacological prevention and treatment of delirium, a reduction in delirium incidence of, for example, absolutely 13.3% [36] or 19% [38] is possible. The incidence of delirium is also described differently in cardiac surgery. There are different frequency methods in data collection with the CAM-ICU from one to three times or more per day. Furthermore, PP possibly has further effects because nonpharmacological delirium prevention and treatment are still being implemented in HDZ NRW [12, 31]. Hence, we might need years for an assured RCT without a guarantee of having certain data for power calculation. That would exceed our time and possibilities for this study, so we decided to carry out a pilot-RCT [44] over the period of one complete year. The data collected can serve as a basis for further research as part of an RCT [14, 43]. In addition, further secondary data analysis is possible and planned.

### Limitations

Our study has limitations. Both ICUs are in the same university hospital. In this way, a contamination bias is possible. On the other hand, ICU 1 and ICU 2 are in different parts of the hospital and have their own nursing teams. This circumstance can reduce the risk of contamination by nurses.

Blinding of nurses in intensive care who carry out primary nursing is not possible.

The CAM-ICU will be taken by especially trained nurses in ICU 1 and ICU 2. All nurses were trained before [31] and the CAM-ICU is a validated assessment for, for example, nurses in ICU. In addition, all nurses will receive another short 1:1 training before this study starts.

Recruitment will take one year from January to December 2023, and we have restricted possible participants. In our opinion, it will be helpful to portray a whole year to test the feasibility of an RCT under real conditions.

Patients must also stay in the ICU for at least 3 days before being included in this study. Relating to protection from delirium, it is necessary to start with nonpharmacological treatment immediately when a patient enters a hospital. As part of a delirium management concept, a catalog of nonpharmacological interventions was implemented in HDZ NRW in 2020 [12, 31]. All departments implemented these measures, including ICU 1 and ICU 2. Furthermore, we evaluated the degree of implementation before the start of PP and after 6 and 12 months of implementation in a separate study, before commencement of this study.

## Supplementary Information

SPIRIT 2013 Checklist

## Abbreviations

CAM-ICU: Confusion Assessment Method for Intensive Care Units
CPOT: Critical Care Pain Observation Tool
HDZ NRW: Heart and Diabetes Center NRW, University Hospital of the Ruhr University Bochum
ICU: Intensive Care Unit
NRS: Numeric Rating Scale
Pilot-RCT: Pilot Randomized Controlled Trial
PN: Primary Nurse/Nursing
PP: Process-responsible nurse/nursing

## Declarations

For this study all investigations on humans described were carried out with the approval of the responsible ethics committee, in accordance with national law and the declaration of Helsinki in 1975 (in the current, revised version). This study is registered at clinicaltrials.gov as NCT05569317.
